# A retrospective study on veterinary antimicrobial use in Nigeria, 2014 to 2017

**DOI:** 10.11604/pamj.2023.45.104.32046

**Published:** 2023-06-23

**Authors:** Dooshima Kwange, Mwapu Dika Ndahi, Olaniran Alabi, Bukar Ali Usman, Peter Umanah, Jens Kirk Andersen, Ayi Vandi Kwaghe

**Affiliations:** 1 Department of Veterinary and Pest Control Services, Federal Ministry of Agriculture and Rural Development, PMB 145, Area 11, Garki, Abuja, Nigeria,; 2 National Agency for Food and Drugs Administration and Control, Plot 1, Isolo Industrial Estate, Lagos, Nigeria,; 3 National Food Institute, Technical University of Denmark, Kemitorvet, Bygning 201, 2800 Kgs, Lyngby, Denmark,; 4 Nigeria Field Epidemiology and Laboratory Training Programme, Zaria, Nigeria

**Keywords:** Retrospective study, imported veterinary antimicrobials, classes of antimicrobials, active ingredients, patterns of antimicrobial use, Nigeria

## To the editors of the Pan African Medical Journal

### Introduction

Global Reliable Data on Antimicrobial Use (AMU) in food animals was estimated at 63,000 tons annually in 2015 and is projected to increase by almost 70% in 2030 [1]. The global program on surveillance of antimicrobial consumption was launched in 2015 by WHO to tackle the problem of a lack of quality antimicrobial use (AMU) data and a standardized methodology for data collection [2]. Data on AMU is fundamental for countries to establish national and local antimicrobial stewardship programs [2]. Antimicrobial resistance (AMR) is a global threat to health and tackling AMR in animals through the aid of AMU information is a means of protecting public health.

The rise in global antimicrobial resistance [3] necessitates the need for the estimation of AMU in the country. This will aid policy decision-making regarding the use of antimicrobials and further give direction regarding the reasons behind specific antimicrobial resistance in the country. In developing nations like Nigeria, data on antimicrobials is underestimated due to the illegal import of drugs as a result of the porosity of our borders. This study aimed at determining the quantity and pattern of veterinary AM used in the country within the study period.

### Methodology

We conducted a retrospective study on the amount of active ingredients of antimicrobial agents intended for use in the animal sector using import data of the country from 2014 to 2017. We obtained data on antimicrobial agents for use in animals from the National Agency for Food and Drug Administration and Control (NAFDAC). Obtained data include; date of importation, name of importing company, name of exporting company, product name, quantity imported. All antimicrobial agents were grouped into the various classes and their active ingredients were calculated. In 2014 and 2015, AM with more than one molecule were calculated as aggregated whereas in 2016 and 2017, AM agents having more than one active ingredient were disaggregated into their respective classes and the amount of active ingredients were calculated. Data for 2014 was for 7 months only.

**Data analyses:** data was collected, collated, analysed, and converted to kilograms as described by Office International des Epizooties (OIE) [4]; active ingredients present in the form of compounds or derivatives, the mass of active entity of the molecules in International Units, were converted to kg [5]. The nomenclature of antimicrobial agents complied with international standards [4].

### Results

Eleven antimicrobial classes were imported for the period under review (2014 to 2017). These include; Tetracyclines (Oxytetracyclines and Chlortetracycline), Fluoroquinolones (Enrofloxacin, Ciprofloxacin and Norfloxacin), Macrolides (Tylosin, Erythromycin and Streptomycin), Penicillin (Amoxycillin and Penicillin), Sulfonamides (Sulfaquinoxalin, Sulfadimidine, Sulfadiazine and Trimethoprim), Polypeptides (Colistin sulphate), Aminoglycosides (Gentamycin and Neomycin), Amphenicols (Florfenicol), Glycopetides (Ancomycin), Pleuromutilins (Tiamulin) and Nitrofurantoin (Nitrofurantoin).

Nineteen antimicrobial had more than one active ingredient; Amoxycol (Amoxycillin and Colistin), Amoxystin (Colistin and Amoxycillin), Dimoxican (Colistin sulphate and Amoxycillin trihydrate), Tylodox (Tylosin and Doxycycline), Doxytyl (Doxycycline and Tylosin), Doxygen, Intergendox (Doxycycline and Gentamycin), Neodox (Doxycycline and Neomycin), Keproceryl (Oxytetracycline, Colistin and Erythromycin), Nemovit, Neotreat, Neo-oxy egg formular (Oxytetracycline and Neomycin), Koleridin (Oxytetracycline and Neomycin), Trisulmix (Trimethoprime and Sulfamethioxine), Colisultrix (Trimethoprime and Colistin sulphate), EST Mix (Erythromycin, Sulfadiazine and Trimethoprime), Sulfa 3 (Sulfathiazole, Sulfadimidine and Sulfamerazine), Sulfavet (Sulphadimidine and Trimethoprime) and Ciprovet (Sulfur and Ciprofloxacin).

A total of 1,392,578 kg (1,392.6 tons) of antimicrobials were imported during the study period (Table 1). The year 2015 had the highest importation of antimicrobials (515,892 kg) then 2017 (338,878 kg) (Table 1). The highest imported class of antimicrobial was tetracycline (629,236 kg) followed by polypeptides (148,974 kg) and macrolides (132,712 kg) (Table 1).

**Table 1:** active ingredients (n=12) of antimicrobials imported to Nigeria, 2014-2017

S/N0	Class of Antimicrobial	2014(Jun-Dec)	2015	2016	2017	Total
		Molecules in kg/ Percentage (%)	Molecules in kg/ Percentage (%)	Molecules in kg/ Percentage (%)	Molecules in kg/ Percentage (%)	Molecules in kg/ Percentage (%)
1	Pleuromutilins	0 (0.00)	0 (0.00)	2 (0.00)	0 (0.00)	2 (0.00)
2	Glycopeptides	24 (0.01)	40 (0.01)	0 (0.00)	0 (0.00)	64 (0.00)
3	Nitrofurantoin	0 (0.00)	0 (0.00)	243 (0.07)	0 (0.00)	243 (0.02)
4	Amphenicols	268 (0.13)	658 (0.13)	0 (0.00)	0 (0.00)	926 (0.07)
5	Penicillins	193 (0.09)	0 (0.00)	6569 (1.99)	3624 (1.07)	10386 (0.75)
6	Sulfonamides	1060 (0.51)	687 (0.13)	6635 (2.01)	11592 (3.42)	19974 (1.43)
7	Fluoroquinolones	5115 (2.47)	3146 (0.61)	13520 (4.09)	3152 (0.93)	24933 (1.79)
8	Aminoglycosides	46 (0.02)	131 (0.03)	37341 (11.29)	9052 (2.67)	46570 (3.34)
9	Macrolides	3349 (0.22)	9798 (1.90)	107775 (32.58)	11790 (3.48)	132712 (9.53)
10	Polypeptides	459 (0.22)	142333 (27.59)	4290 (1.30)	1892 (0.56)	148974 (10.70)
11	Aggregated	188339 (90.99)	190219 (36.87)	0 (0.00)	0 (0.00)	378558 (27.18)
12	Tetracyclines	8147 (3.94)	168880 (32.74)	154433 (46.68)	297776 (87.87)	629236 (45.18)
Total		207000 (100.00)	515892 (100.00)	330806 (100.00)	338878 (100.00)	1392578 (100.00)

* aggregated; antimicrobial agents with more than one molecule regardless of the antimicrobial class were calculated as aggregated (2014-2015)

### Discussion

This report is similar to the global trend of AMU which tends to be decreasing [1]. During the four years, tetracycline (45.18%) accounted for most of the imported AM with almost 300 tons in 2017 followed by polypeptides (10.70%) and macrolides (9.53%) similar to a study in south-western Nigeria where stating tracycline's (33.6%) as the leading antimicrobial used in livestock production over the period of study [6]. Several reports from Africa, UK and OIE revealed tetracycline group as the most used antibiotic [7-9].

Nitrofurantoin and pleuromutilin were only imported in 2016. The use of antimicrobial agents in animal health has a potential of affecting human health through drug residues, as a result, Nitrofuran and Amphenicols were banned for use in livestock feed in many countries, including Nigeria (NAFDAC) [10]. This also indicates that the Nigerian government is adhering to the banning of these drugs hence the absence of their importation in subsequent years.

## Conclusion

Data on AMU in Nigeria for the study period was 1,392,578 kg (1392.6 tons). In Nigeria, AMU data is underestimated because antimicrobials produced locally, those purchased through informal markets and other sources such as veterinary clinics/hospitals and farms were not captured. This study demonstrates the need for collecting such data to improve on the current data collection system.
